# Austin County Medical Society

**Published:** 1893-09

**Authors:** 


**The** Austin County Medical Association was organized in Bellville, on July 18th last, with the following officers elected for one year: W. R. P. Thompson, of Nelsonville, President; M. DeCausey, of Sealy, Vice-President; A. H. Schenk, of Kenney, Secretary and Treasurer. It meets on the third Tuesday of each month, in Bellville.

---

**A recent** invention is that of an apparatus designed for registering rises in temperature. A small metallic bulb is half filled with ether and sealed by a corrugated cover. When the temperature rises, the ethereal vapor straightens out the corrugations of this cover, and is made to close an electric circuit which works a bell. It is suggested that such an apparatus be adjusted to the axilla of each patient in a hospital, and attached to a numbered bell in the intern's room, so that any sudden febrile access may be at once known.—*Ex.*

